# Experimental evolution-induced transcriptome and phenotype responses of *Drosophila melanogaster* to novel thermal environments

**DOI:** 10.1242/jeb.251365

**Published:** 2025-10-10

**Authors:** Dau Dayal Aggarwal, Prachi Mishra, Yashvant Patel, Manvender Singh, Vijendra Sharma, Abraham B. Korol, Pawel Michalak

**Affiliations:** ^1^ Department of Biochemistry, University of Delhi South Campus, New Delhi, Delhi 110021, India; ^2^ Department of Zoology, Banaras Hindu University, Varanasi, Uttar Pradesh 221005, India; ^3^ Department of Biotechnology, UIET, MD University, Rohtak, Haryana 124001, India; ^4^ Department of Biomedical Sciences, University of Windsor, ON, Canada, N9B 3P4; ^5^ Institute of Evolution, University of Haifa, Haifa 3498838, Israel; ^6^ Department of Evolutionary and Environmental Biology, University of Haifa, Haifa 3498838, Israel; ^7^ Edward Via College of Osteopathic Medicine, Monroe, LA 71203, USA; ^8^ Center for One Health Research, Virginia-Maryland College of Veterinary Medicine, Blacksburg, VA 24061, USA

**Keywords:** Thermal stress, Genomic adaptation, Transcriptome profiling, Physiological assays, Thermal regime-specific patterns

## Abstract

Thermal stress imposes significant challenges on organisms, influencing cellular functions, morphology and survival. This study investigates the transcriptomic and phenotypic adaptations of *Drosophila melanogaster* populations subjected to constant high-temperature (HT) and fluctuating-temperature (FT) regimes over 80 generations in experimental evolution settings. RNA sequencing identified 1288 and 1152 differentially expressed genes in HT and FT populations, respectively, relative to the baseline population. Multiple gene ontology (GO) terms, including chromatin organization, nucleosome assembly, nucleic acid binding and polytene chromosome band formation, were enriched under both regimes, suggesting shared adaptive pathways. A weighted gene co-expression network analysis (WGCNA) revealed mitochondrial function and protein homeostasis as central to thermal adaptation, with HT populations showing enrichment of DNA repair and FT populations exhibiting enrichment of RNA processing and translation regulation-related terms. Phenotypic assays demonstrated increased heat tolerance, accelerated development and prolonged longevity in evolved populations, highlighting parallel as well as thermal regime-specific adaptive responses. This study emphasizes the complexity of transcriptomic–phenotypic adaptations to thermal stress in new environments.

## Teaser

**Summary:** Experimental evolution in *Drosophila melanogaster* under constant and fluctuating thermal regimes reveals parallel and regime-specific transcriptomic and phenotypic adaptations, highlighting polygenic and regulatory changes that shape responses to environmental stress.

## INTRODUCTION

Heat stress disrupts homeostasis at multiple biological levels, from the cellular to the organismal scale, affecting nearly all living species (Angilletta, 2009; Gross, 2006; Nagano et al., 2019). This challenge is particularly acute for ectotherms, which cannot regulate internal body temperature independently of ambient conditions (Angilletta, 2009; Hoffmann, 2010). With the ongoing shifts in global climate patterns, the need for ectotherms to develop significant genomic adaptations to survive in increasingly dynamic thermal environments has become more pressing (McCulloch and Waters, 2023). While previous studies have demonstrated that thermal adaptations are often polygenic, involving complex gene networks (Angilletta, 2009; Barghi et al., 2019), a substantial gap remains in our understanding of whether naturally fluctuating temperatures over just a few dozen life cycles can drive adaptive responses.

The genus *Drosophila* serves as a model for investigating evolutionary responses to thermal stresses because of its extensive thermal tolerance (Hoffmann et al., 2003; Kellermann et al., 2009) and well-characterized genetic architecture (Mackay, 2004). Previous studies have primarily utilized two approaches to investigate thermal adaptive responses in *Drosophila*: comparative analysis of natural populations enduring contrasting temperatures and experimental evolution conducted under controlled laboratory conditions. Natural population observations indicate that *Drosophila* species inhabiting arid, high-temperature environments demonstrate enhanced resistance to heat stress compared with those from cooler regions (Hübner et al., 2013; Parkash et al., 2009), a trend also observed across the continents (Hoffmann, 2010; Hoffmann et al., 2003). This pattern shows considerable local adaptation to thermal gradients despite continuous gene flow, with clinal differences in thermal tolerance emphasizing the role of natural selection (Hoffmann and Parsons, 1997).

At the molecular level, *Drosophila* studies have primarily focused on the role of heat shock proteins (HSPs) and heat shock factors (HSFs) in conferring thermal adaptations (Feder and Hofmann, 1999; Hoffmann et al., 2003). Genome-wide transcriptome analyses have elucidated gene expression patterns across different geographic populations, revealing significant differences linked to geographic origin and local temperature regimes (Hutter et al., 2008; Müller et al., 2011). Further, tropical and temperate *Drosophila* populations display unique transcriptome profiles, revealing a notable trend of downregulation across various gene sets in response to non-native temperatures (Hutter et al., 2008). Comparative genomic studies emphasize species-specific patterns of clinal variation, showing that *Drosophila melanogaster* exhibits greater differentiation and isolation by distance compared with *Drosophila simulans* (Zhao et al., 2015), likely attributable to variation in migration patterns and the stability of clinally varying loci.

Experimental evolution combined with resequencing (‘evolve and resequence’, E&R) provides a reliable approach for analysing the genetic foundations of adaptive traits in a controlled and replicable setting (Schlötterer et al., 2014, 2015). In recent decades, E&R studies have pinpointed genetic loci linked to traits including hypoxia tolerance (Zhou et al., 2011), desiccation resistance (Kang et al., 2016), starvation **(**Hardy et al., 2018**)** and thermal tolerance (Orozco-Terwengel et al., 2012; Schlötterer et al., 2015). E&R has advanced our understanding of adaptive traits, yet pinpointing causative genes for stress adaptation remains challenging. This complexity stems from the multifaceted nature of stress, marked by intricate interactions among physiological systems and the fact that experimental evolution, though powerful under controlled conditions, may not fully capture the environmental heterogeneity and overlapping selective pressures characteristic of natural ecosystems. Nevertheless, E&R remains a valuable tool for dissecting evolutionary dynamics under defined selective regimes, providing insights that are often difficult to obtain in complex natural environments (Burke et al., 2010; Schlötterer et al., 2015).

Bridging the gap between laboratory findings and natural evolutionary processes has thus become a key focus in recent years (Kellermann et al., 2015; Phillips and Burke, 2021). Hsu and co-workers (2021) demonstrated that transcriptomic signatures associated with thermal responses identified through E&R experiments substantially overlap with those observed in natural populations of *D. melanogaster*. This concordance suggests that lab-based evolution studies can uncover core mechanisms of thermal adaptation, though findings must be contextualized within the multifactorial selective pressures and ecological variability of natural habitats (Phillips and Burke, 2021).

This study investigates how *D. melanogaster* adapts to a constant high temperature (31°C) and a fluctuating thermal regime (22°C/31°C) over 80 generations. Using RNA sequencing, we compared the transcriptomes of base (B), high-temperature (HT) and fluctuating-temperature (FT) evolved lines to identify differentially expressed genes and pathways involved in these adaptive responses. Our findings reveal both shared and distinct gene expression patterns in HT and FT environments. Additionally, weighted gene co-expression network analysis (WGCNA) identified gene modules enriched for these common and unique responses, offering deeper insights into the gene networks underlying thermal adaptation. We also assessed whether the observed gene expression changes align with those reported in previous studies, highlighting potential parallels between experimental and natural evolutionary trends. Furthermore, analyses of phenotypic, physiological and stress-related traits indicate a strong concordance in adaptive responses across HT and FT lines, suggesting that similar underlying mechanisms may (at least partially) drive adaptation in the two conditions, despite differences in temperature regimes.

## MATERIALS AND METHODS

### Study design and experimental evolution

Depending on geographic location, *Drosophila melanogaster* Meigen 1830 populations inhabit a wide range of thermal environments. In the midland region of Solan, Himachal Pradesh (India), summer daytime temperatures typically range from 28 to 33°C, while night-time temperatures drop to 17–22°C. In contrast, lowland regions such as Chandigarh, Ambala and Rohtak experience consistently higher daytime temperatures (often exceeding 35°C) and warmer nights (24–28°C) (India Meteorological Department, 2015). Notably, Indian Meteorological Department data for September 2016 (time of sample collection) indicate diel fluctuations in Chandigarh between approximately 22°C at night and 33°C during the day. These natural thermal profiles guided our experimental design to investigate how realistic environmental variability influences transcriptomic and phenotypic adaptation.

Wild *D. melanogaster* flies were collected in September 2016 from Chandigarh, India (altitude: 321 m, latitude: 30.7°N; average temperature: 29°C), and 1328 isofemale (IF) lines were established from the collected samples. These IF lines were then pooled to create a mass culture maintained in the laboratory for six generations to reduce potential phenotypic plasticity effects before initiating the experimental treatments. Approximately 500 gravid females were randomly selected from this mass culture and allowed to oviposit in nine large culture jars (24×12 cm), ensuring adequate space and food per larva. This formed the basis of our selection lines: base (B1–B3), high-temperature (HT1–HT3) and fluctuating temperature (FT1–FT3). Each replicate was subdivided into three sub-replicates (∼650–700 larvae each), with an effective population size of ∼1500 flies per replicate per generation. To standardize larval density, egg laying was restricted to a 6–8 h period on fresh food, and jars were visually inspected for consistency. Each sub-replicate was cultured independently. All flies were maintained on freshly prepared cornmeal–yeast–agar medium (cornmeal: 38 g l^−1^, yeast: 20 g l^−1^, agar: 7.6 g l^−1^, glucose: 35 g l^−1^) supplemented with propionic acid and methylparaben to prevent microbial growth. Fresh food was provided twice per generation, first to newly eclosed adults (1–2 days old), and again in oviposition jars for the next generation. Populations were maintained under non-overlapping generations. To maintain a large pool of standing genetic variation and minimize the effects of random genetic drift, flies from all sub-replicates were pooled at each generation.

**Fig. 1.**
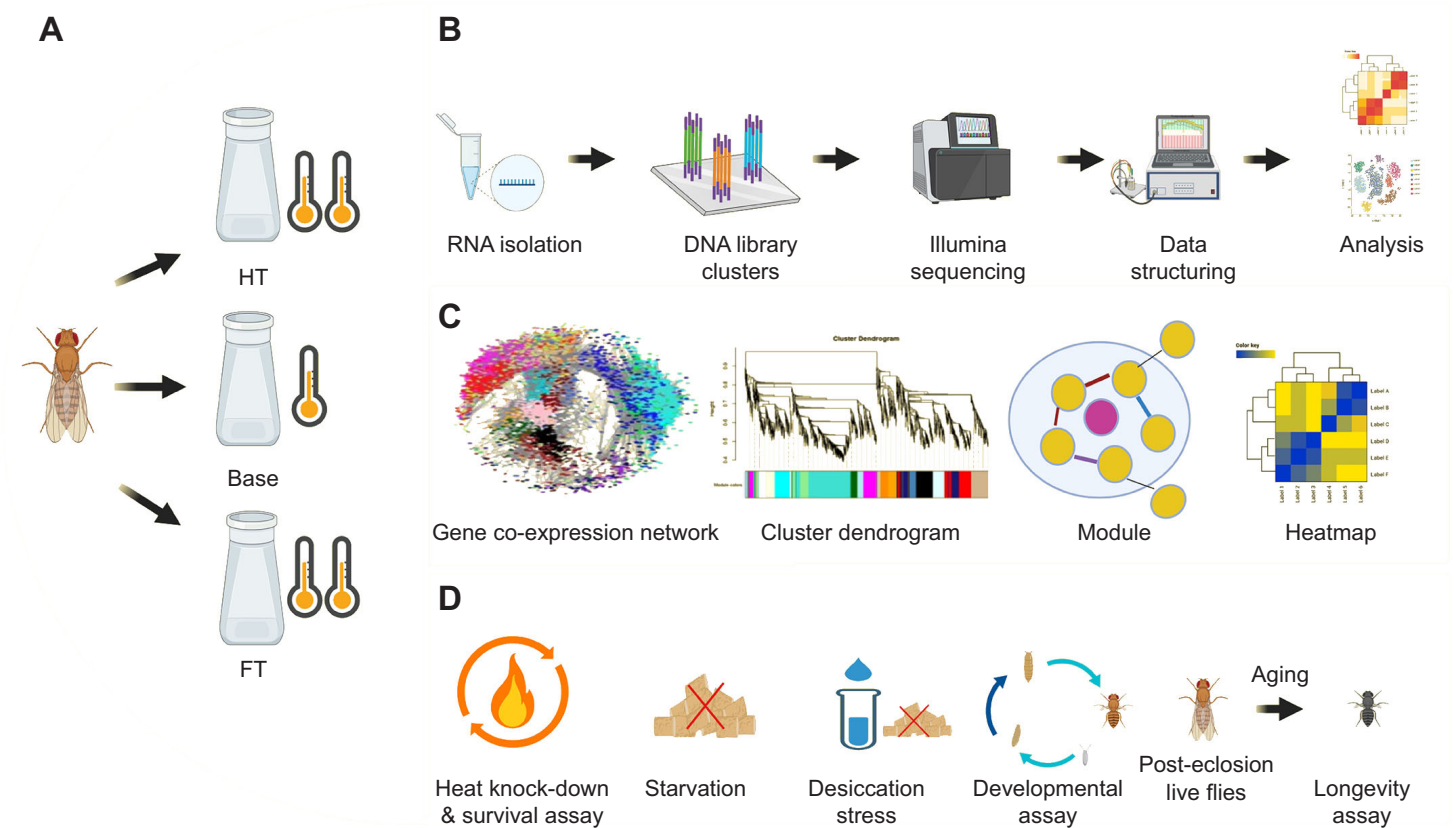
**A schematic presentation of the experimental design.** (A) Experimental evolution (∼5 years): *Drosophila melanogaster* populations were subject to high-temperature (HT) and fluctuating-temperature (FT) growth regimes for >80 generations, while the base (B, control) groups were maintained at ambient temperature. (B) Transcriptome profiling: RNA samples were collected from all three variants, and Illumina sequencing was performed to retrieve the transcriptome signatures. (C) Transcriptome data were used for weighted gene co-expression network analysis (WGCNA). (D) To assess the correlated changes in physiological traits, a series of physiological and phenotypic assays were conducted on individuals from all three groups. These assays included heat knockdown, percentage survival, starvation tolerance and desiccation tolerance, duration of development and longevity.

Base population (B) replicates were maintained at a constant temperature of 22±0.5°C. The HT replicates were reared at 30.5±0.5°C for over 80 non-overlapping generations. In contrast, the FT replicates were exposed to a thermal regime alternating between ambient temperature (22±0.5°C) during the night and elevated temperatures (30.5±0.5°C) during the day, mimicking the diel temperature fluctuations experienced by *Drosophila* populations in their native habitats. Temperature cycles were independently validated using a Lascar EL-USB-2 temperature data logger placed in a representative culture jar to confirm they matched programmed conditions. All cultures were maintained under a 12 h light:12 h dark cycle using a programmable digital timer, with relative humidity kept between 60% and 70%, as regularly monitored using a hygrometer. To account for faster development under elevated temperatures, experimental progression was tracked by generation number rather than calendar time. This ensured that all treatments, despite differences in real-time duration, completed over 80 discrete generations, allowing direct evolutionary comparisons. To minimize environmental and transgenerational effects across the experimental replicates, we employed a common garden experimental design in which both the HT and FT populations were reared at 22±0.5°C for a few generations before we conducted RNA sequencing and phenotype assays. This approach ensured that the findings primarily reflect genetic accommodation in gene expression and phenotypic traits rather than short-term phenotypic plasticity. Also, RNA-Seq samples for all experimental groups were collected at Zeitgeber time ZT4–ZT5 (4–5 h after lights-on) to minimize short-term environmental effects and normalize any shifts in circadian phase across regimes. A schematic overview of the experimental design is shown in Fig. 1.

### High-throughput transcriptomic profiling

A total of 180 individual virgin females (20 from each replicate population) were randomly selected for RNA extraction using the TRIzol™ Plus RNA Purification Kit (Thermo Fisher Scientific). Each RNA sample was evaluated using a NanoDrop spectrophotometer and gel electrophoresis, confirming the presence of distinct 18S and 28S ribosomal RNA bands and thereby validating RNA integrity. Following quality control, samples from each replicate were pooled at equal concentrations (150 ng µl^−1^ per sample), resulting in nine pooled samples. The pooled RNA samples were subjected to high-throughput paired-end sequencing using the Illumina NovaSeq 6000 platform, with libraries prepared using the TruSeq Stranded Total RNA Library Prep Kit following rRNA depletion via the Ribo-Zero Plus kit to enhance mRNA detection.

Raw RNA-Seq reads were first assessed for quality using FastQC (version 0.11.3), then trimmed with Trimmomatic (version 3.4; Bolger et al., 2014) to ensure high-quality data for downstream analysis. The *D. melanogaster* genome (BDG6.32) served as the reference for read alignment using the STAR software package (version 2.7; Dobin et al., 2013). Summary statistics of the obtained reads can be found in Table S1. On average, each pooled sample yielded approximately 18 million reads, covering a total of 23,932 genes. Gene expression levels were normalized with RSEM (version 1.3.3; Li and Dewey, 2011) to account for differences in sequencing depth and ensure consistency in downstream comparisons. To reduce statistical noise and focus on genes with sufficient expression levels, those with a count matrix sum (B and HT/FT) less than 20 were eliminated to increase analytical robustness.

EdgeR (version 3.22.5; Robinson et al., 2010) was employed for differential gene expression analysis, comparing gene expression profiles of the evolved populations (HT and FT) with that of their ancestral base (B) population. Gene expression was modelled using the equation *y*=Evolution+ε (Hsu et al., 2021), where *y* represents the normalized expression level of each gene, ‘Evolution’ denotes one of three states (HT, FT or B) and ε accounts for the unexplained variation or random error. Two contrasts were performed: (i) comparing the average responses of HT and FT replicates against their shared base population replicates (referred to as concordant evolution) and (ii) comparing HT and FT replicates with each other to identify regime-specific evolutionary patterns. Gene ontology (GO) enrichment analysis was performed using the TopGO R package (version 2.48.0; Alexa et al., 2006). *P*-values were adjusted for multiple testing using the Benjamini and Hochberg false discovery rate (FDR) correction method (Haynes, 2013).

### Gene co-expression network analysis

WGCNA was conducted to identify temperature-adapted gene modules, following the approach described by Hsu et al. (2021). We used log-transformed counts per million normalized gene expression data from all genes in our dataset. WGCNA was performed using the R WGCNA package (Langfelder and Horvath, 2008), starting with Pearson's correlation coefficients to measure gene co-expression. These correlation coefficients were then raised to a power β to generate an adjacency matrix that satisfied scale-free topology requirements, with 500 bootstrap iterations ensuring robust network construction and parameter selection. By emphasizing stronger correlations in the adjacency matrix, weaker associations were effectively filtered out. The next step involved computing a topological overlap matrix (TOM) to compare connectivity among gene pairs. This TOM guided hierarchical clustering, enabling the construction of the co-expression network and the identification of gene modules. Following guidelines provided by the WGCNA package creators, we identified β=6 as optimal for our data. GO enrichment analysis was performed using TopGO to elucidate each module's biological significance, which identified statistically enriched functional categories and pathways within each module. This analysis provided deeper insights into the specific biological processes associated with temperature adaptation.

### Comparison with published data

We compared our common responsive genes (CRGs), exclusive responsive genes (ERGs) and baseline comparative differential analyses with findings from previous studies on low- and high-temperature field habitats in America (Zhao et al., 2015; Project ID: SRP047141), and Africa versus Europe (Hutter et al., 2008) on gene expression. We also compared our expression patterns with those reported by Manenti et al. (2018) and Hsu et al. (2021) to examine potential parallels with earlier thermal experimental evolution studies.

### Phenotypic assays

Each replicate line was subdivided into ten sub-replicates, with 100 females randomly assigned to each sub-replicate, resulting in 1000 individuals per replicate.

#### Heat knockdown and percentage survival assays

Groups of ten 3 day old, post-eclosion females were placed in individual 5 ml glass vials, which were then immersed in a water bath maintained at 39°C. The time (in minutes) required for each fly to be knocked down was recorded. Likewise, flies were exposed to 39°C for 90 min, followed by a 20 h recovery period. The percentage of flies capable of walking afterwards was used to measure survival.

#### Starvation tolerance

Starvation tolerance was assessed by recording the time to death from lack of food. Groups of ten 3 day old post-eclosion females were placed in dry plastic vials (37 mm×100 mm) containing a foam sponge with 2 ml of water and 2 mg of sodium benzoate. These vials were maintained at a constant temperature of 22±0.5°C. The number of immobile flies was recorded at regular intervals: 3 times on the first day, 8 times on the second day and approximately 20 times per day until all flies had succumbed to starvation stress.

#### Desiccation tolerance

Desiccation resistance was measured as the time taken for flies to reach lethal dehydration (LT90) under dry air conditions. Groups of ten 3 day old post-eclosion females were placed in dry plastic vials containing 2 g of silica gel, with the gel covered by a disc of foam. These vials were transferred to a desiccator chamber (Secador electronic desiccator cabinet; www.tarson.in), with a relative humidity of 0–5%. The number of immobile flies was recorded at 1 h intervals, and the time to lethal desiccation effect (LT90) was calculated.

#### Duration of development and longevity

Three-day-old females were mated once and placed individually in culture vials (37 mm×100 mm) containing cornmeal yeast–agar medium. Each female was allowed to lay eggs for 60 min at 22°C. The vials were regularly inspected, and the duration of development from egg to third instar larvae and pupae was recorded for each population (B, HT and FT). Following eclosion, virgin flies were transferred to fresh vials containing standard food medium. The flies were transferred to new vials every fourth day, and instances of mortality were recorded. Longevity was calculated for each group, defined as the number of days from eclosion to death.

#### Statistical analyses

Phenotypic trait data were summarized by calculating mean values along with their corresponding standard deviations (s.d.). To assess differences in physiological traits across temperature regimes (HT, FT and B), we employed a generalized linear model (GLM), with replicates nested within the respective base and experimental groups to account for within-group variation. All statistical analyses and figure generation were performed using custom scripts written in Python and R. The analyzed data are presented in the main figures and tables, and additional supporting datasets are provided in Tables S1–S7.

## RESULTS

### Gene expression divergence

**Fig. 2.**
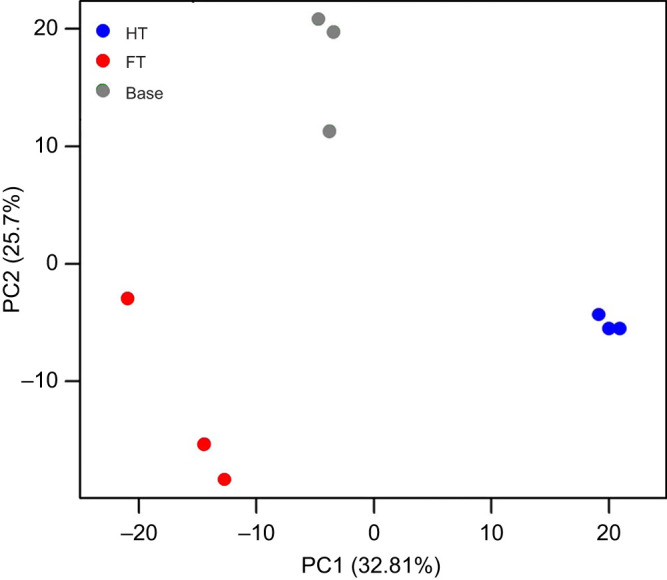
**Principal component analysis (PCA) of gene expression profiles across base, HT and FT evolved lines.** Each point represents a biological replicate (grey, base; blue, HT; red, FT). PCA was performed on EdgeR-processed RNA-Seq data. PC1 and PC2 denote the first two principal components, capturing the majority of variance in the dataset. Distinct clustering of HT and FT lines, separated from the base group, reflects significant transcriptional divergence driven by long-term thermal selection. The spatial separation between HT and FT clusters further indicates lineage-specific transcriptomic responses to constant versus fluctuating thermal regimes, despite their shared ancestral origin.

PCA of the gene expression data revealed clear distinctions among the conditions (Fig. 2). The base replicates clustered closely, suggesting a consistent gene expression profile, whereas the HT and FT lines were distinctly separated from the base group along PC1 and PC2. This clear separation emphasizes the substantial transcriptomic divergence driven by long-term exposure to different thermal environments. This result supports the hypothesis that distinct temperature conditions select for unique gene expression profiles that contribute to adaptive responses.

### Baseline comparative differential analysis

**Fig. 3.**
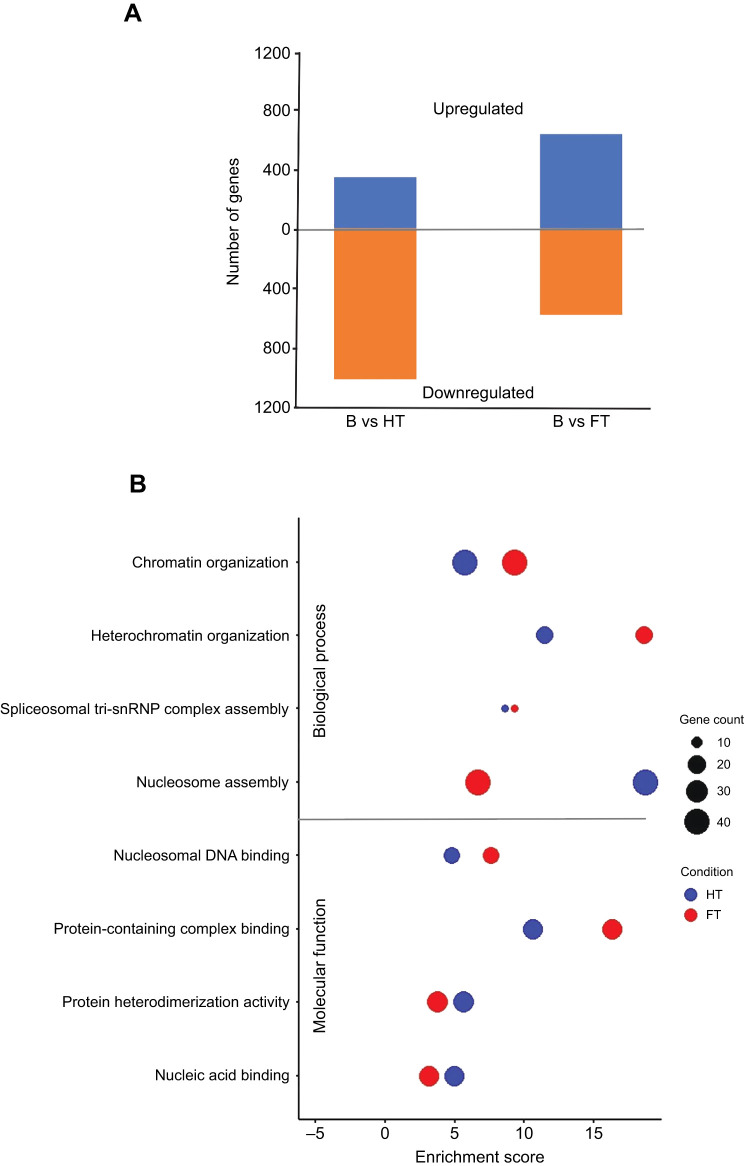
**Differential gene expression analysis and gene ontology (GO) enrichment in HT and FT lines.** (A) Bar plot shows the number of upregulated (blue) and downregulated (orange) genes identified in each regime versus the base group. (B) Bubble plot presents enriched GO terms (Biological Process and Molecular Function) among differentially expressed genes identified with EdgeR in HT (blue) and FT (red) lines, analysed using TopGO. The *x*-axis denotes enrichment scores, reflecting statistical significance, while bubble size indicates the number of genes associated with each term. Both HT and FT lines exhibit enrichment in regulatory functions, with differing enrichment magnitudes and gene associations indicating distinct molecular adaptations shaped by their respective thermal environments.

We identified 1288 genes differentially expressed under HT (326 upregulated, 962 downregulated) and 1152 genes under FT (600 upregulated, 552 downregulated) compared with the base line (Fig. 3A; *P*<0.05; Table S1), thus accounting for approximately 10% of the *Drosophila* coding genome. Multiple GO terms, including DNA binding, chromatin organization, nucleosome assembly and polytene chromosome band formation, were enriched (*P*<0.05) under both HT and FT conditions (Fig. 3B), suggesting shared mechanisms of thermal adaptation. Nonetheless, distinct gene enrichment patterns indicate variation in the specific genomic responses between HT and FT.

### Concordant and divergent gene responses in a network context

**Fig. 4.**
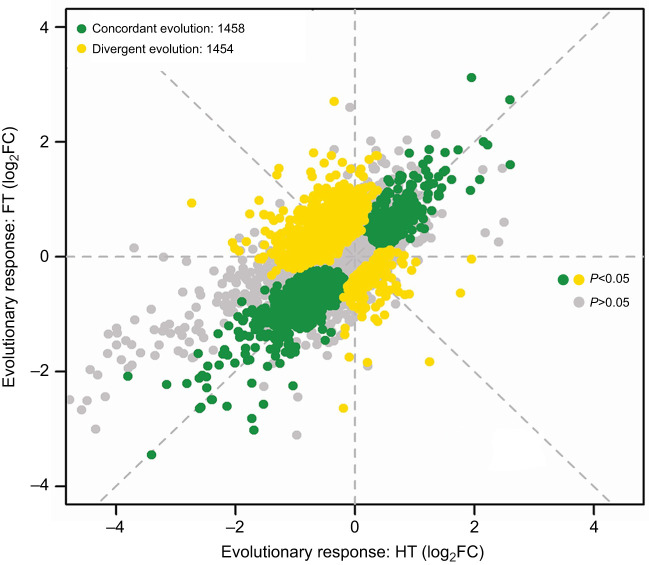
**Scatter plot showing expression changes in HT- and FT-evolved replicates relative to the base population.** Each point represents a gene identified by EdgeR differential expression analysis, with expression changes (log_2_ fold-change, FC) in HT lines on the *x*-axis and in FT lines on the *y*-axis. Genes with similar expression shifts in the two regimes cluster along the diagonal (blue), indicating parallel transcriptional responses. Genes showing regime-specific expression changes deviate from this diagonal (yellow), reflecting divergent transcriptional adaptations shaped by distinct thermal selection pressures.

We identified 1108 genes showing similar expression changes (408 upregulated, 700 downregulated) under both HT and FT conditions, referred to as CRGs. Additionally, 1104 genes exhibited divergent expression patterns, labelled as ERGs, with 326 genes upregulated in HT and 962 downregulated in FT conditions (Fig. 4; Tables S2 and S3). Using WGCNA, we detected gene modules enriched for CRGs and ERGs, reflecting the polygenic and interconnected nature of thermal adaptation at the network level. Notably, upregulated CRGs were significantly enriched in modules 2 and 8 [Fisher's Exact Test (FET) test odds ratio: OR≥12.48; *P*<0.05], while downregulated CRGs were prominent in modules 1 and 17 (FET test OR≥11.81; *P*<0.05; Table 1). These CRG modules implicate shared adaptive pathways, suggesting that certain gene networks provide broad-spectrum thermal resilience. HT ERGs were concentrated in modules 9, 14 and 19 (FET test OR≥1.45; *P*<0.05), while FT ERGs dominated modules 3, 6, 7, 15 and 16 (FET test OR≥3.44; *P*<0.05; Table S4, sheets 1–20). This divergence in module composition indicates that the transcriptomic responses are not solely parallel but also specialized, highlighting how different thermal regimes exert distinct selective pressures that shape specific gene networks, aligning with our findings of temperature-specific pathway enrichment.

**Table 1.** Co-regulatory modules (top 20) from weighted gene co-expression network analysis (WGCNA) and functional enrichment of adaptive candidate genes and gene ontology (GO) terms from this study

Module ID	No. of genes	Top term	Evolution laboratory	*P*	Odds ratio
Module0	29	Peptidyl-serine phosphorylation			
Module1	837	rRNA processing	CRG-downregulated	2.20E−16	11.81096
Module2	538	GPI anchor biosynthetic process	CRG-upregulated	2.20E−16	12.48663
Module3	528	Regulation of axonogenesis	ERG-FT	2.20E−16	5.203777
Module4	401	Kinetochore assembly			
Module5	394	Transmembrane transport			
Module6	369	Chemical synaptic transmission	ERG-FT	2.20E−16	37.71301
Module7	367	Axon guidance	ERG-FT	2.20E−16	5.108713
Module8	361	Melanotic encapsulation of foreign target	CRG-upregulated	2.20E−16	12.69867
Module9	323	Glutathione metabolic process	ERG-HT	0.07986	1.450235
Module10	319	Positive regulation of JNK cascade			
Module11	315	Glutathione metabolic process			
Module12	313	Transmembrane transport			
Module13	302	Indole-containing compound metabolic process			
Module14	295	Mitochondrial translation	ERG-HT	2.20E−16	24.82034
Module15	264	Proteolysis	ERG-FT	2.20E−16	4.181347
Module16	227	Motor neuron axon guidance	ERG-FT	7.41E−15	3.441972
Module17	216	RNA processing	CRG-downregulated	2.20E−16	11.92096
Module18	212	Protein folding			
Module19	206	Vesicle tethering	ERG-HT	0.02017	1.855985
Module20	199	DNA endoreduplication			

CRG, common responsive genes; ERG, exclusive responsive genes; HT, high-temperature evolved specific; FT, fluctuating-temperature evolved specific.

### GO analysis of concordant and divergent genes

Within the CRG-upregulated category, enriched GO terms related to mitochondrial function, such as mitochondrial translation (GO:0032543) and mitochondrial translational elongation (GO:0070125), underscore the critical role of mitochondria in energy production and cellular resilience under thermal stress (Table S5). These results align with the broader adaptive significance of mitochondrial efficiency for maintaining cellular homeostasis during prolonged thermal exposure (Angilletta, 2009; Hoffmann and Parsons, 1997). The enrichment of homologous chromosome segregation (GO:0045143) and proteasome-mediated ubiquitin-dependent protein catabolic process (GO:0043161) highlights the importance of genetic stability and protein quality control in thermal tolerance. This observation reinforces that these conserved pathways are essential for adaptation to high and fluctuating temperatures, even though the specific gene drivers differ between the conditions (as seen in ERG-specific modules). In the CRG-downregulated category, enriched terms such as positive regulation of circadian sleep/wake cycle (GO:0045938) and activation of GTPase activity (GO:0090630) imply that thermal stress influences circadian rhythm regulation and cellular signalling processes. The downregulation of circadian genes may reflect an adaptive response rather than an experimental artefact, as RNA samples were collected at the same Zeitgeber time (ZT4–ZT5) across treatments to minimize circadian variation.

Within the ERG-FT category, we observed enrichment in RNA processing (GO:0006396) and cytoplasmic translation (GO:0002181) terms, emphasizing the importance of RNA metabolism and protein production in adapting to variable thermal conditions. Conversely, ERG-HT genes highlighted DNA repair mechanisms, with heat shock-mediated polytene chromosome puffing (GO:0035080), double-strand break repair via homologous recombination (GO:0000724) and response to ionizing radiation (GO:0010212), indicating a focus on maintaining genetic integrity at high temperatures. These findings suggest that while CRGs provide general thermal resilience, ERGs are fine-tuned to address specific challenges posed by constant versus fluctuating temperatures, reflecting the nuanced nature of genomic–transcriptomic adaptation. Although these GO terms provide a wider lens on the biological processes involving responsive genes, transcriptome data alone are limited in fully capturing and contextualizing these complex interactions.

### Differential expression of stress-related genes

Baseline comparative differential analysis revealed a significant upregulation of *Hsp70Aa*, *Hsp70Ab* and *Hsp70Ba* under HT conditions (*P*=4E−02 to 7E−11; Table S1), indicating a heightened heat shock response that is crucial for coping with extreme thermal stress. Conversely, no notable changes in HSP expression were observed under FT conditions, suggesting that adaptation in the FT lines involves alternative stress management mechanisms, corroborated by the enrichment of RNA processing genes in the ERG-FT category. While several other HSP family members showed expression variation under both HT and FT regimes, these changes did not meet the threshold for statistical significance. Additionally, the differential expression of genes (*GstD*-*10*, *Gst*-*13* and *Dip-B*) in the glutathione pathway between HT and FT conditions highlights their involvement in oxidative stress regulation, which is likely central to thermal stress adaptation.

### Gene expression comparisons with other studies

**Fig. 5.**
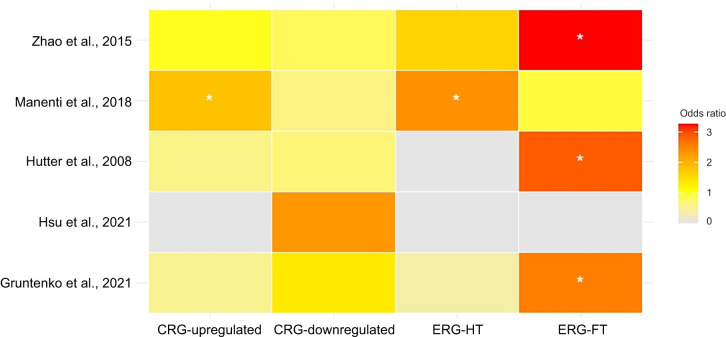
**Comparative enrichment analysis of gene categories across studies.** A colour-coded representation showing odds ratios (colour gradient) for gene categories from this study (CRG-upregulated, CRG-downregulated, ERG-HT, ERG-FT) compared with findings from other studies. The colour intensity reflects the magnitude of the odds ratio, with red indicating higher ratios and yellow indicating lower ratios. Asterisks denote statistically significant values (*P<0.05).

In comparing gene expression patterns across multiple categories, including CRG-upregulated, CRG-downregulated, ERG-HT, ERG­FT, B versus HT and B versus FT, several notable observations emerged, illustrating both significant overlaps and clear distinctions between our results and those reported in earlier studies (Fig. 5; Tables S6 and S7).

#### CRG-upregulated category

A significant overlap was identified with the gene expression data from Manenti et al. (2018) (FET test, OR=1.83; *P*=0.03), indicating that many of the genes upregulated in both HT and FT conditions in our study were also enriched in their dataset. However, comparisons with other studies, particularly those examining natural populations (Hutter et al., 2008; Zhao et al., 2015), revealed no significant enrichment. This suggests that the genes concordantly upregulated in our experiments may be more specifically aligned with our controlled conditions than with natural settings.

#### CRG-downregulated category

No strong associations emerged with any other dataset. Although some shared patterns of gene downregulation between our study and Hsu et al. (2021) may exist, the *P*-value was statistically non-significant.

#### ERG-HT category

The most significant enrichment was observed with the data from Manenti et al. (2018) (FET test OR=2.38; *P=*0.007).

#### ERG-FT category

Genes responding exclusively to FT conditions exhibited significant enrichment, particularly with the datasets from Zhao et al. (2015) (FET test OR=3.26; *P*=2.20E−16) Gruntenko and co-workers (2021) (FET test OR=2.57; *P*=0.001) and Hutter et al. (2008) (FET test OR=2.87; *P*=6E−04).

#### B versus HT and B versus FT

With the Manenti et al. (2018) dataset, genes from our B versus HT (FET test OR=2.09; *P*=7E−04) and B versus FT (FET test OR=1.96; *P*=3E−03) comparisons showed significant enrichment, implying a strong overlap of thermally responsive genes in baseline differential analyses between our study and that study. Similarly, B versus HT (FET test OR=6.41, *P*=8.3E−12) and B versus FT (FET test OR=7.24; *P*=6.02E−13) gene sets were highly enriched in the Gruntenko et al. (2021) dataset. In the Hutter et al. (2008) dataset, genes from our B versus HT comparison exhibited significant enrichment (FET test OR=2.93; *P*=7E−04). In contrast, no significant enrichment was observed for B versus FT (FET test OR=1.54; *P*=0.17). This finding suggests that genes responding to constant high-temperature conditions align more closely with those identified by Hutter and colleagues (2008). Lastly, the comparisons of our B versus HT (*P*=0.08) and B versus FT (*P*=0.07) datasets with that of Hsu et al. (2021) approached significance, indicating a potential parallel in gene expression patterns, though these trends did not reach statistical significance.

### Physiological assays

**Fig. 6.**
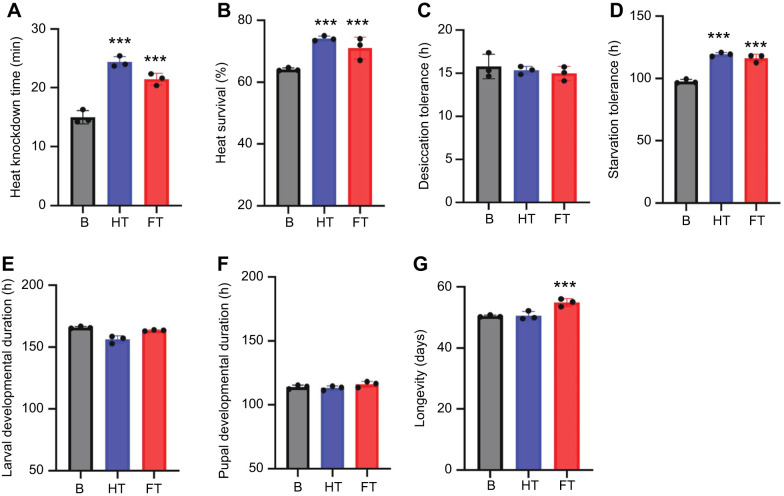
**Comparative physiological performance across base, HT and FT lines.** (A) Heat knockdown time, (B) heat survival percentage, (C) desiccation tolerance, (D) starvation tolerance, (E) larval developmental duration, (F) pupal developmental duration and (G) adult longevity. Both HT and FT lines show significantly enhanced heat resistance relative to the base population (A,B). Starvation tolerance (D) is significantly improved in both HT and FT lines compared with the base line. No significant differences were observed in desiccation tolerance (C), larval development time (E) or pupal development time (F) across treatments. Notably, FT lines exhibit significantly extended adult lifespan compared with both HT and base lines (G). Data are presented as means±s.e.m.; asterisks denote significant differences from base lines (***P<0.001). Each point represents a biological replicate.

We observed several key differences among the base, HT and FT lines (Fig. 6). Both HT (*F*=332.06, *P*=1.01E−10) and FT (*F*=174.98, *P*=1.01E−10) groups showed a significant increase in heat knockdown time, indicating enhanced heat tolerance. Survival after heat stress was also higher in HT (*F*=63.69, *P*=1.01E−8) and FT (*F*=159.05, *P*<1.01E−10), with no notable changes in desiccation tolerance. Starvation tolerance significantly increased in HT (*F*=166.32, *P*<1.01E−10) and FT (*F*=193.66, *P*<1.01E−10), indicating greater resistance to food deprivation. HT lines showed accelerated development (*F=*70.09, *P*<1.01E−8), while FT lines exhibited no such change. FT groups had increased longevity (*F*=43.02, *P*=2E−7), while HT lines did not differ from the base line in longevity (GLM ANOVA; Table 2).

**Table 2.** The results of the GLM model, Type II (nested design, with replicates nested within control/experimental groups), showing the comparison of physiological traits between base (B) and HT, as well as base and FT replicates

Trait	d.f.	B vs HT			B vs FT		
		MS	*F*	*P*	MS	*F*	*P*
Heat knockdown time (min)	1	1312.18	332.06	<1.0E−10	620.75	174.98	<1.0E−10
	4	10.66	2.69	1.04E−01	11.40	3.214	1.9E−02
	54	3.95			3.55		
Heat survival time (min)	1	714.2	63.69	<1.0E−8	1490.0	159.05	<1.0E−10
	4	64.20	5.73	6.4E−04	4.6	0.49	7.4E−01
	54	11.21			9.4		
Desiccation tolerance time (h)	1	9.94	2.916	9.3E−02	2.91	0.883	3.5E−01
	4	13.39	3.927	7.1E−03	11.07	3.354	1.5E−02
	54	3.41			3.30		
Starvation tolerance (h)	1	5236.2	166.32	<1.0E−10	7047.1	193.66	<1.0E−10
	4	62.9	2.00	1.0E−01	26.8	0.74	5.7E01
	54	31.5			36.4		
Larval developmental duration (h)	1	1411	70.09	<1.0E−8	86	6.1	1.6E−02
	4	48	2.39	6.2E−02	5	0.3	8.1E−01
	54	20			14		
Pupal developmental duration (h)	1	64.1	1.80	1.8E−01	10.4	10.4	2.3E−01
	4	36.7	1.03	3.9E−01	103.1	25.8	5.7E−01
	54	35.5			2427.3	45.0	
Longevity (days)	1	0.30	0.05	8.1E−01	296.61	43.02	2.E−07
	4	10.5	1.67	1.7E−01	8.60	1.25	3.0E−01
	54	6.31			6.92		

MS, mean square; d.f., degrees of freedom.

## DISCUSSION

### Adaptive gene expression in novel temperature environments

Our study demonstrates significant divergence in gene expression between *D. melanogaster* lines adapted to HT and FT regimes compared with baseline populations after 80 generations of thermal adaptation. We identified 1288 differentially expressed genes in HT populations and 1152 in FT populations, highlighting the polygenic complexity of thermal adaptation. Consistent with prior research, our findings infer the critical role of stress-response mechanisms, including the upregulation of HSPs and genes involved in protein stabilization, DNA repair and degradation. Overlapping and distinct patterns of gene expression between HT and FT populations suggest that thermal regimes exert unique selective pressures, driving specialized adaptive responses while maintaining certain shared mechanisms for resilience across thermal conditions.

GO enrichment analysis of biological process terms, focusing on baseline comparative differential expression, revealed both shared and distinct patterns between thermal regimes. HT and FT populations demonstrated similar gene expression responses in biological pathways related to nucleosome assembly (GO:0006334), chromatin organization (GO:0006325) and proteolysis (GO:0006508). These processes are crucial for sustaining genomic stability through chromatin regulation and ensuring protein homeostasis via targeted protein degradation in response to thermal stress (Angilletta, 2009; Hübner et al., 2013; Pessa et al., 2024). Nonetheless, FT populations exhibited a notable downregulation of genes associated with circadian rhythms and energy metabolism, potentially enhancing physiological adaptability to varying thermal conditions by reducing dependence on rigid, time-dependent mechanisms. This downregulation may reflect shifts in metabolic efficiency, allowing organisms to conserve energy under stress and reactivate key pathways when temperatures improve (Chown et al., 2004; Neven, 2000). Conversely, the gene expression patterns observed in HT populations suggest that prolonged exposure to elevated temperatures necessitates the upregulation of genomic maintenance systems to preserve cellular integrity. While such expression profiles are likely shaped by thermal regimes, laboratory-imposed constraints – such as diet, density and photoperiod – may further interact with these stressors to influence adaptive gene expression. Acknowledging these interactions is important for interpreting the overlapping and divergent transcriptomic responses observed across HT and FT lines.

In addition to biological process-level enrichment, molecular function categories revealed both shared and regime-specific signatures. Common enrichment of DNA binding (GO:0003677), nucleosomal DNA binding (GO:0031492), structural constituent of chromatin (GO:0030527) and protein heterodimerization activity (GO:0046982) suggests chromatin remodelling and regulation of protein complexes as core thermal responses across HT and FT lines. HT-specific enrichment of oxidoreductase activity (GO:0016705), haeme binding (GO:0020037) and iron ion binding (GO:0005506) reflects a heightened need for redox balance and mitochondrial regulation under persistent heat stress. In contrast, FT populations showed greater enrichment of serine-type endopeptidase activity (GO:0004252), pointing to dynamic protein turnover mechanisms that support flexibility in fluctuating environments. Together, these molecular function terms complement the biological process findings, indicating that thermal adaptation involves a coordinated response across levels of gene function from regulatory networks and chromatin accessibility to enzyme activity and proteostasis.

To ensure that the observed gene expression differences reflected evolved responses rather than short-term plasticity or non-genetic influences, all populations were maintained under a common garden design for over six generations in identical, sterile conditions prior to RNA sequencing and phenotypic assay. Standardized diets, controlled incubators and strict hygiene protocols minimized microbial variability. Large population sizes also buffered against genetic drift and the accumulation of deleterious alleles (Wright, 1931). While it is impossible to entirely rule out transgenerational epigenetic effects or contributions from microbial communities, our observation of consistent transcriptomic shifts and phenotypic responses across multiple independently evolved lines strongly argues against such non-genetic factors being primary drivers of the observed adaptive differences. Nonetheless, we acknowledge this limitation and note that additional metagenomic profiling or epigenomic assays would be necessary to exclude these factors. Additionally, to minimize circadian confounds, all lines were kept on a 12 h:12 h light:dark cycle and sampled at the same Zeitgeber time (ZT4–ZT5) after common-garden rearing. However, residual evolved differences in circadian phase between HT and FT lines may still contribute to the observed transcriptomic and phenotypic contrasts; resolving this will require time–series sampling across multiple circadian times and/or assays under constant conditions.

### Shared and divergent gene expression patterns reflect adaptive strategies

Our results indicate the complexity of thermal adaptation, with both shared and unique pathways driving responses to high and fluctuating temperature environments. Among the 1108 CRGs, those involved in mitochondrial function and proteasome-mediated protein degradation were central to adaptation under HT and FT conditions, reflecting a core mechanism for preserving cellular homeostasis during thermal stress**.** This supports findings from the literature highlighting mitochondrial efficiency and protein quality control as critical components of thermal adaptation across multiple species (Hood et al., 2018; Koch et al., 2021). However, the divergence observed in ERGs points to distinct adaptive strategies. HT populations exhibited upregulation of DNA repair-related genes, likely as a mechanism to prevent DNA damage and maintain genomic stability under constant high temperatures (Lupu et al., 2004; Shaposhnikov et al., 2015). In contrast, FT populations showed enriched GO terms related to RNA processing and cytoplasmic translation, suggesting a flexible gene expression response including dynamic RNA editing and alternative splicing (Anduaga et al., 2019; Rieder et al., 2015) suited to variable conditions. Similarly, Manenti and co-workers (2015, 2016) reported that long-term evolution under fluctuating temperatures in *D. simulans* resulted in genomic divergence without corresponding changes in plasticity, with adaptation driven primarily through shifts in trait means. While we did not directly assess plasticity, the enriched RNA processing and translation pathways observed in our FT populations are indicative of transcriptomic flexibility through mechanisms such as dynamic RNA editing and alternative splicing, suggesting a regulatory strategy favouring flexible gene expression over evolved plasticity.

### Gene co-expression networks highlight functional significance

WGCNA revealed different gene modules associated with CRGs and ERGs, indicating coordinated gene expression in response to thermal stress. CRG-upregulated modules (2 and 8) were enriched for mitochondrial function genes, lysosomal transport and proteoglycan biosynthesis, implying that energy production and cellular maintenance are adaptive responses in HT and FT settings. In contrast, ERG-specific HT-enriched modules are dominated by DNA repair and genomic stability pathways such as double-strand break repair, emphasizing the need for genetic integrity in adjusting to persistent high temperatures. Other processes enriched under HT include the unfolded protein response and stress-activated protein kinase signalling cascade, which mitigate cellular stress caused by extreme thermal conditions. FT-specific modules were enriched in RNA processing and protein synthesis pathways, suggesting that changing temperature conditions need higher gene expression flexibility and post-transcriptional control. WGCNA shows that functional categories enable complex system-level adaptive responses. This supports the idea that integrated networks of co-regulated genes drive thermal adaptation and associated responses.

### HSP responses in HT and FT regimes

The upregulation of HSPs in HT populations supports their established role in mitigating thermal damage by preventing protein misfolding and aggregation (Angilletta, 2009; Feder and Hofmann, 1999; Hoffmann et al., 2003). Our results demonstrate a pronounced heat stress response, with significant upregulation of *Hsp70Aa*, *Hsp70Ab* and *Hsp70Ba* genes under HT conditions, consistent with previous findings suggesting that chronic heat stress affects HSP profiles. In contrast, FT populations did not exhibit a similar HSP-driven response, as also observed by Manenti et al. (2018), indicating that fluctuating temperatures may not impose a sustained requirement for protein stabilization. Studies in *Caenorhabditis elegans* have demonstrated that HSPs are induced temperature specifically, with significant upregulation occurring primarily under acute heat stress conditions (Rubio-Tomás et al., 2024). This is consistent with previous observations indicating that HSP expression is triggered mainly by intense, short-term heat exposure (Feder and Hofmann, 1999), whereas fluctuating temperature regimes tend to favour broader stress responses rather than temperature-specific pathways such as HSPs only. While this pattern in our data initially suggested a reduced reliance on temperature-specific stress responses in FT lines, we acknowledge that fluctuating environments may also select for alternative preparatory mechanisms.

### Translating laboratory results to natural environments

Laboratory studies have significantly advanced our understanding of thermal adaptation by isolating key variables under controlled conditions, enabling precise insights into evolutionary mechanisms. Yet, their simplified environments may yield adaptive patterns that only partially reflect nature's complex selective landscapes.

Notably, CRG-upregulated genes in our data showed substantial overlap with laboratory-based datasets, such as the microarray study by Manenti et al. (2018), yet exhibited minimal correspondence with gene expression profiles from natural populations – including the study by Zhao et al. (2015), which analysed both *D. melanogaster* and *D. simulans* using Illumina HiSeq, and the microarray-based study of Hutter et al. (2008) conducted at a constant 22°C. In contrast, ERG-FT genes demonstrated strong enrichment with multiple natural population datasets (Hutter et al., 2008; Zhao et al., 2015), suggesting that responses to fluctuating environments may better reflect naturally occurring adaptive patterns. Similarly, B versus HT and B versus FT comparisons showed significant alignment with both laboratory-based and field studies (Hutter et al., 2008; Manenti et al., 2018), highlighting the broader relevance of baseline differential responses. Additionally, the HT and FT populations in our study showed some alignment – although not statistically significant (*P*≈0.05) – with that of Hsu et al. (2021), which employed a similar experimental evolution framework using daily temperature cycling and an Illumina-based sequencing platform.

These mixed patterns likely reflect a combination of biological and technical factors, including interspecific comparisons, founder population backgrounds, thermal regime differences and transcriptomic platform biases. Such sources of variation must be acknowledged when interpreting overlaps across studies. Nonetheless, when comparisons are based on closely matched parameters, meaningful and reproducible trends can still emerge. We therefore advocate that future experimental evolution studies incorporate ecologically realistic designs, genetically diverse founders and standardized transcriptomic approaches to enhance cross-study comparability.

### Correlated changes in physiological traits

In this study, both HT and FT populations showed increased heat tolerance, as shown by longer heat knockdown times and higher post-stress survival rates. For HT populations, accelerated development appears advantageous in constant high-stress environments by minimizing exposure during vulnerable developmental stages, a correlated response frequently noted in stable thermal adaptation studies (Parsons, 1997). Conversely, FT populations exhibited increased longevity, which may offer a survival advantage in variable conditions (Colinet et al., 2018), correlating with the theory that intermittent stress selects for traits promoting extended lifespan (Parsons, 2007). The rapid development in HT populations may conserve energy by shortening sensitive life stages, thereby limiting exposure to constant stress. In contrast, FT populations likely benefit from traits enhancing survival across environmental fluctuations, where extended lifespan serves as a buffer against unpredictable stressors.

Additionally, neither HT nor FT lines exhibited changes in desiccation tolerance, yet both showed significantly enhanced starvation tolerance. Previous studies using thermal performance curves for *Drosophila* traits, such as fecundity (Klepsatel et al., 2023), lifespan (Miquel et al., 1976) and metabolic rate (Kellermann et al., 2019), demonstrate that thermal adaptation is trait specific and dependent on both exposure duration and developmental stage. Such complexity implies that evolutionary responses to thermal stress involve multifaceted adaptations, with individual traits evolving independently while balancing correlated fitness trade-offs. Overall, these results highlight how adaptive responses to high temperatures engage complex interactions among physiological, genetic and life-history traits, elucidating that organisms navigate environmental changes across diverse thermal landscapes.

In conclusion, this study establishes how distinct thermal environments drive adaptive responses in *D. melanogaster*. Through transcriptome profiling and physiological analyses, we have uncovered both shared and environment-specific signatures of thermal responses. Lines exposed to constant high temperature showed upregulation of pathways supporting genomic stabilization, while populations evolving under fluctuating conditions exhibited shifts in translational regulation and RNA processing, without clear induction of classical stress-response mechanisms. Physiological assays confirmed enhanced heat tolerance, improved starvation resistance and altered longevity, directly linking thermal adaptation to the evolution of stress-related phenotypes. While earlier studies have explored thermal adaptation using artificial cycles, our work advances the field by employing realistic diel fluctuations, a genetically diverse founder population and parallel assessment of transcriptomic changes alongside key physiological traits, offering complementary insights into both molecular and phenotypic aspects of thermal adaptation. Long-term selection over 80 generations under constant and fluctuating thermal regimes defines a unique experimental evolution framework, offering novel insights into genomic and physiological adaptation to sustained environmental stress – captured at this scale for the first time.

## Supplementary Material

10.1242/jexbio.251365_sup1 Supplementary information

Table S1. Baseline comparative differential expression analysis: *Base vs. HT* (Sheet 1)and *Base vs. FT* (Sheet 2). Summary of RNA-seq read quality and alignment statistics for all samples (Sheet 3).

Table S2. Likelihood ratio test results (Sheet 1) and differentially expressed environmentally responsive genes (CRGs) (Sheet 2).

Table S3. Likelihood ratio test results (Sheet 1) and differentially expressed environmentally responsive genes (ERGs) (Sheet 2).

Table S4. WGCNA module assignments and associated gene ontology (GO) terms (Sheets 1-20).

Table S5. Enrichment analysis of CRG and ER genes (sheets 1-5)

Table S6. Differential expression data from previous studies used for comparative analysis (sheets 1-5).
