# Supplementary material for: Experimental evolution-induced transcriptome and phenotype responses of Drosophila melanogaster to novel thermal environments
Source: J Exp Biol. 2025 Oct 10;228(19):jeb251365. doi: 10.1242/jeb.251365 (PMC12539207; doi:10.1242/jeb.251365)
Supplement: Supplementary information [file jexbio-228-251365-s1.pdf]

Table S1. Baseline comparative differential expression analysis: *Base vs. HT* (Sheet 1) and *Base vs. FT* (Sheet 2). Summary of RNA-seq read quality and alignment statistics for all samples (Sheet 3).

Available for download at
https://journals.biologists.com/jeb/article-lookup/doi/10.1242/jeb.251365#supplementary-data

Table S2. Likelihood ratio test results (Sheet 1) and differentially expressed candidate regulatory genes (CRGs) (Sheet 2).

Available for download at
https://journals.biologists.com/jeb/article-lookup/doi/10.1242/jeb.251365#supplementary-data

Table S3. Likelihood ratio test results (Sheet 1) and differentially expressed environmentally responsive genes (ERGs) (Sheet 2).

Available for download at
https://journals.biologists.com/jeb/article-lookup/doi/10.1242/jeb.251365#supplementary-data

Table S4. WGCNA module assignments and associated gene ontology (GO) terms (Sheets 1–20).

Available for download at
https://journals.biologists.com/jeb/article-lookup/doi/10.1242/jeb.251365#supplementary-data

Table S5. Enrichment analysis of CRG and ER genes (sheets 1-5)

Available for download at
https://journals.biologists.com/jeb/article-lookup/doi/10.1242/jeb.251365#supplementary-data

Table S6. Differential expression data from previous studies used for comparative analysis (sheets 1-5).

Available for download at
https://journals.biologists.com/jeb/article-lookup/doi/10.1242/jeb.251365#supplementary-data

Table S7. Fisher's exact test comparative analysis between present and previous studies. Comparative analysis of expression profiling from present data versus previous studies

	**Comparative analysis of expression profiling: Present data versus previous studies**						
References		CRG_up	CRG_down	ERG_HT	ERG_FT	BvsHT	BvsFT
Zhao et al., [19]	OR	1.05	0.86	1.61	3.26	0	0
	P value	0.51	0.80	0.18	2.20E-16	1	1
Manenti et al., [39]	OR	1.83	0.62	2.38	0.97	2.09	1.96
	P value	0.03	0.94	0.007	0.57	0.0007	0.003
Gruntenko et al., [40]	OR	0.55	1.35	0.41	2.57	6.41	7.24
	P value	0.87	0.26	0.91	0.001	8.37E-12	6.02E-13
Hutter et al., [18]	OR	0.60	0.69	0	2.87	2.93	1.54
	P value	0.84	0.82	1	0.0006	0.0007	0.17
Hsu et al., [30]	OR	0	2.31	0	0	2.51	2.36
	P value	1	0.08	1	1	0.08	0.07

OR: Odds ratio

Journal of Experimental Biology • Supplementary information
